# Assent and Negation Language and Emotional Experiences in Married Couples

**DOI:** 10.1093/geroni/igaf122.2103

**Published:** 2025-12-31

**Authors:** Lillian Fu, Tabea Meier, Claudia Haase

**Affiliations:** University of California, Irvine, Evanston, Illinois, United States; Northwestern University, School of Education and Social Policy, Evanston, Illinois, United States; Northwestern University, Evanston, Illinois, United States

## Abstract

Emotional processes in marital interactions predict late-life well-being and health outcomes, but few studies have examined how words that convey validation or invalidation may shape the emotional climate of marital interactions. In a laboratory-based dyadic interaction study with 49 married middle-aged couples (N = 98 individuals; ages 21-65) from diverse socioeconomic and racial/ethnic backgrounds, we examined links between assent (e.g., “mhm”, “yes”) and negation (e.g., “no”, “never”) language and emotional experiences. Couples engaged in 10-minute videotaped conflict and pleasant conversations. Then, they individually reported on the intensity of their subjective negative and positive emotional experiences. The relative frequency of assent and negation words during each conversation was calculated using the Linguistic Inquiry and Word Count software. Linear mixed models revealed that individuals’ assent language and partners’ assent language were both associated with lower levels of negative emotional experiences. Moreover, partners’ negation language was associated with higher levels of own negative emotional experiences, and a conversation interaction effect revealed that this association was driven by conflict conversations. Results generalized across gender and remained stable when controlling for age, socioeconomic status, negation/assent word frequency, and total word count. These findings highlight the dyadic interplay between spouses’ use of assent and negation words and emotional experiences. Implications for dyadic interaction research with couples across the lifespan and large-scale text analysis will be discussed.

